# Social Isolation, Loneliness and Well-being in the Covid-19 Crisis: A Look at Nursing Home Residents in Luxembourg

**DOI:** 10.1093/geroni/igaa057.3501

**Published:** 2020-12-16

**Authors:** Isabelle Albert, Martine Hoffmann, Anna Kornadt, Elke Murdock, Josepha Nell

**Affiliations:** 1 University of Luxembourg, Esch-sur-Alzette, Luxembourg; 2 RBS Center fier Altersfroen, Itzig, Luxembourg; 3 University of Luxembourg, Esch-sur-Alzette, Diekirch, Luxembourg

## Abstract

During the COVID-19 crisis, older adults, in particular those with underlying health conditions, were at a special risk for severe illness and mortality, and efforts were made to shield them from exposure to the virus. While measures of physical distancing and reduction of in-person contacts were necessary to prevent contraction, they hit residents of care settings particularly hard since visits from family and friends were banned and the risk for loneliness and social isolation increased. In the present study, we therefore gave the voice to nursing home residents and focused on their perceived loneliness and subjective well-being during the crisis. We were both interested in difficulties but also in personal resources and resilience factors that might protect older adults from negative mental health outcomes and help to maintain subjective well-being. A sample of N = 76 residents in care homes in the Grand Duchy of Luxembourg were interviewed by use of a standardized questionnaire during July and August 2020. Participants reported on their loneliness and life satisfaction during the crisis, on their self-regulatory strategies as well as on personal and social resources (e.g. self-efficacy, generativity, social support). Data will be analyzed by use of regression analysis to predict loneliness and well-being by risk and protective factors. Results will be discussed applying a life-span developmental and systemic perspective to understand the mutual interplay of individual, social and institutional resources to mitigate negative side effects of protective measures on care home residents.

